# Histomorphometric Study of Non-critical Bone Defect Repair after Implantation of Magnesium-substituted Hydroxyapatite Microspheres

**DOI:** 10.1055/s-0044-1787768

**Published:** 2024-09-04

**Authors:** Jacqueline de Azerêdo Silva, George Gonçalves dos Santos, Iorrana Índira dos Anjos Ribeiro, Ana Maria Guerreiro Braga da Silva, Isabela Cerqueira Barreto, Marcos Almeida Matos, Maurício Andrade Barreto, Fúlvio Borges Miguel

**Affiliations:** 1 Centro de Medicina Hiperbárica do Nordeste (CMHN), Salvador, BA, Brasil; 2 Centro de Ciências da Saúde (CCS), Universidade Federal do Recôncavo da Bahia (UFRB), Santo Antônio de Jesus, BA, Brasil; 3 Programa de Pós-Graduação em Processos Interativos dos Órgãos e Sistemas (PPGPIOS), Faculdade Adventista da Bahia (FADBA), Cachoeira, BA, Brasil; 4 Centro de Ciências Agrárias, Ambientais e Biológicas (CCAAB), Universidade Federal do Recôncavo da Bahia (UFRB), Cruz das Almas, BA, Brasil; 5 Instituto de Ciências da Saúde (ICS), Universidade Federal da Bahia (UFBA), Salvador, BA, Brasil; 6 Escola Bahiana de Medicina e Saúde Pública (EBMSP), Salvador, BA, Brasil

**Keywords:** biomaterials, bone and bones, bone regeneration, hydroxyapatite, magnesium

## Abstract

**Objective** The present study aims to analyze histomorphometrically the repair of a non-critical bone defect after implantation of hydroxyapatite (HA) microspheres substituted by magnesium (Mg).

**Methods** Thirty rats were distributed into 3 experimental groups, evaluated at 15 and 45 days postoperatively: HAG (bone defect filled with HA microspheres); HAMgG (bone defect filled with HA microspheres replaced with 1 mol% Mg), and CG (bone defect without implantation of biomaterials).

**Results** After 15 days, the biomaterials filled the entire defect extent, forming a new osteoid matrix between the microspheres. In the CG, this neoformation was restricted to the edges with the deposition of loose connective tissue with reduced thickness. At 45 days, new bone formation filled almost the entire extension of the bone defect in the 3 groups, with statistically significant osteoid deposition in the CG despite the reduced thickness compared with the HAG and HAMgG. The groups with biomaterial implantation displayed a more abundant osteoid matrix than at 15 days.

**Conclusion** The biomaterials studied showed biocompatibility, osteoconductivity, and bioactivity. The Mg concentration in the substituted HA did not stimulate more significant bone formation than HA without this ion.

## Introduction

Bone tissue bioengineering, an emerging, interdisciplinary, and multidisciplinary area, has gained prominence in recent years due to the technical-scientific advances achieved and the increasingly growing biomedical and socioeconomic demands in today's society. Researchers in this area have applied the principles of biological and health sciences, chemistry, physics, and engineering to developing and improving regenerative techniques and biomaterials capable of restoring or improving the function and aesthetics of compromised tissues and organs. 1 2 3 4

To define the applicability of these biomaterials, they shall present physical-chemical, biological, and morphological properties similar and compatible with living tissues to act as three-dimensional (3D) scaffolds assisting tissue regeneration or working as suitable replacements for damaged or lost tissues and organs. Among the various biomaterials currently available, calcium phosphate bioceramics (CaP) represent a widely researched and used class in bone regenerative techniques. 1 Among these materials, synthetic hydroxyapatite (HA) has stood out in recent decades due to its biocompatibility, similarity with biological apatite, bioactivity, osteoconductivity, non-immunogenicity, and enabling cellular events observed during tissue regeneration, such as angiogenesis and osteogenesis. 1 2 3 4 However, this ceramic presents slow biodegradation and bioresorption rates after *in vivo* implantation, asynchronous to the bone regeneration mechanism. 1 2 3 4 Furthermore, it is a rigid and brittle material, which can remain in the implantation site for months and even years, depending on the synthesis and processing method. 4 5

Given this, one of the main objectives of researchers in this area has been to improve the properties of synthetic HA and modify the characteristics of this material to enhance the tissue response after *in vivo* implantation. Among the different methods available to carry out these modifications, HA-hexagonal-structure isomorphic substitutions have shown satisfactory results 3 6 due to the effects on the physical properties of the material, observed by changes in network parameters, crystalline structure, morphology, solubility, and thermal stability compared to unsubstituted HA. 7

In these cases, other metals, such as zinc (Zn), strontium (Sr), fluorine (F), manganese (Mn), and magnesium (Mg), can replace calcium (Ca). 3 4 6 Magnesium has attracted great scientific interest, considering that, among other properties, it participates in the homeostasis of bone tissue and, together with other minerals, is fundamental to the mechanism of bone regeneration, stimulating bone formation, through the activation of osteoblasts and inhibiting resorption, through action on osteoclasts. 8 9 10 11

Despite these possibilities, the physicochemical properties, biological behavior, and regenerative capacity of CaP substituted with Mg still require further studies to ensure the effectiveness of the techniques used during the synthesis and processing of these biomaterials. Therefore, this study evaluated the repair of non-critical bone defects after implantation of Mg-substituted HA microspheres in the rats' calvaria.

## Materials and Methods

### Biomaterials

The biomaterials evaluated in this study have been synthesized, processed, and sterilized in the Biomaterials Laboratory (LABIOMAT, in the Portuguese acronym) of the Brazilian Center for Physical Research (Centro Brasileiro de Pesquisas Físicas, CBPF). The synthesis, processing, and characterization are described in Santos et al. 4

### Surgical Procedures

**Fig. 1:**
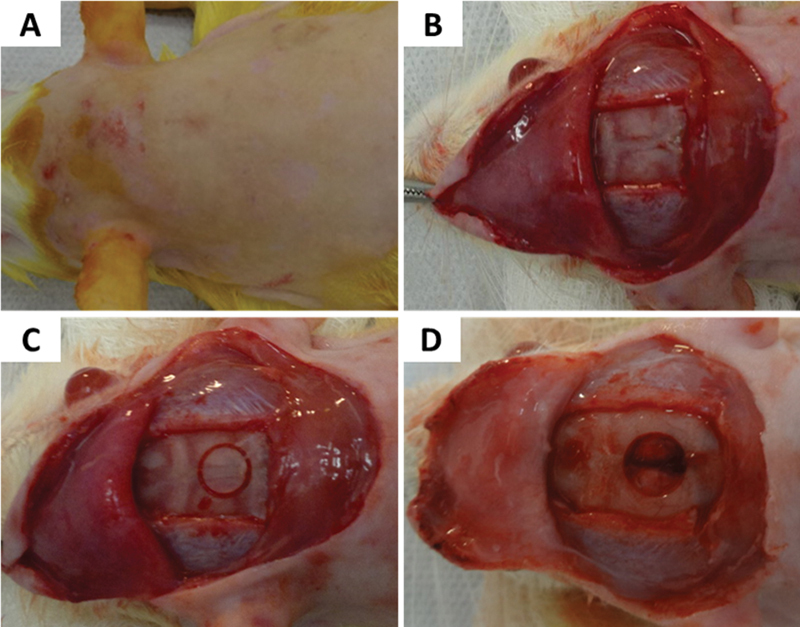
Surgical steps for creating a non-critical bone defect. ( **A** ) Calvarial region after trichotomy and antisepsis; ( **B** ) flap reflected after a semilunar bi-coronal skin incision to expose the bone tissue; ( **C** ) demarcation of the bone defect in the median portion of the calvaria; and ( **D** ) non-critical bone defect created.

This study was performed in the bioterium of Universidade Estadual de Feira de Santana (UEFS) after approval by the Ethics Committee on Animal Use (CEUA, in the Portuguese acronym) of Escola Bahiana de Medicina e Saúde Pública, protocol 02/2013. Thirty adults male *Wistar* rats, with body weight between 350 and 400g, were randomly distributed to form 3 experimental groups, with 5 animals each: HAG (defect filled with HA microspheres group); HAMgG (bone defect filled with MgHA microspheres group), CG – control group (bone defect without biomaterial), evaluated 15 and 45 days after surgery. The surgical technique used was the same as described by Miguel et al. 12 and illustrated by Santos et al. 2 However, it is worth highlighting that in these studies, the bone defect was approximately 8.0 mm in diameter, while in this study, it was about 5.0 mm 13 ( Fig. 1 ).

### Histological Processing and Histomorphological Analysis

At the biological points of 15 and 45 days, the animals have been euthanized with a lethal ketamine and xylazine dose. Then, the upper portion of the calvaria was removed, the soft tissues were discarded, and the specimens were fixed in 4% buffered formaldehyde for 7 days. After this step, they were decalcified in 5% nitric acid for 2 hours, embedded in paraffin, and cut into 5.0-µm thick slices. The histological sections obtained were stained with hematoxylin-eosin (HE) and subsequently examined by standard light microscopy (DM1000–Leica Microsystems, Wetzlar, Germany) for histomorphological and morphometric analysis. A digital camera (DFC 310 FX – Leica Camera AG, Wetzlar, Germany) coupled to a standard light microscope (DM1000–Leica) and the software QWin 3.1 (Leica) measured the area of the newly formed mineralized matrix in the 3 experimental groups. Subsequently, statistical analysis was carried out based on the mean and standard deviation to obtain the *p* -value, with a significance level of 5% ( *p* < 0.05), using the analysis of variance (ANOVA).

## Results

### Histomorphological Analysis

**Fig. 2:**
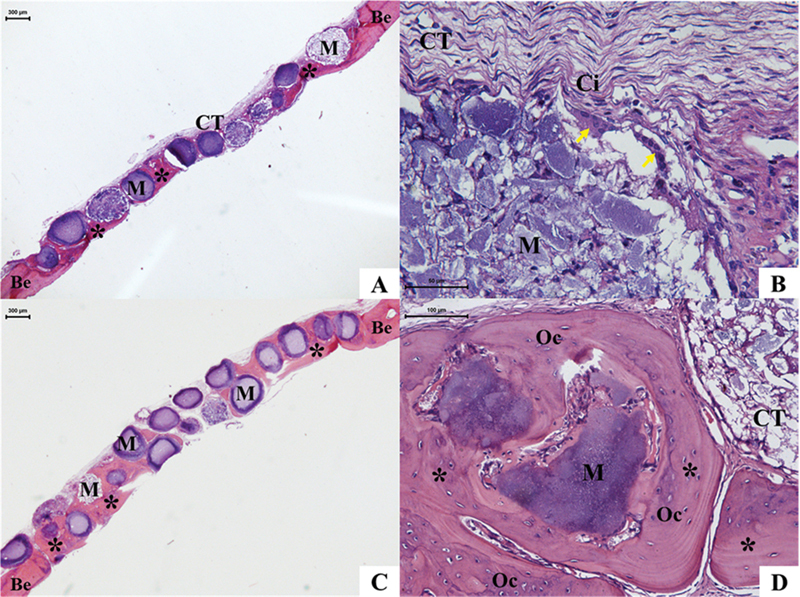
Photomicrograph of HAG – 15 and 45 days. Note microspheres (M) distributed in a mono or double layer, with the formation of an osteoid matrix (*), in almost the entire extension of the defect, with the presence of osteocytes (OCs) and organized in concentric bone lamellae; loose connective tissue (CT) in the residual area of the bone defect, with the presence of mononuclear inflammatory cells (Ci) and multinucleated giant cells (yellow arrow). Abbreviations: Be, bone edge; HE, hematoxylin and eosin. 15 days ( **A** , **B** ). 45 days ( **C** , **D** ).

At 15 days, in the HAG, neoformation of an osteoid matrix was observed associated with the bone edges and surrounding some microspheres, which were predominantly organized in a monolayer and occupied the entire extension of the bone defect, maintaining the thickness proportional to the edges ( Fig. 2A ). Active osteocytes and osteoblasts were observed associated with this matrix. The formation of connective tissue and mild chronic inflammation with mononuclear inflammatory infiltrate and multinucleated giant cells, mainly around the microspheres, was noted between the mineralized areas and the microspheres ( Fig. 2B ).

At the biological point of 45 days, the microspheres in the HAG were mainly distributed in multilayers. The newly formed osteoid matrix was more evident than at 15 days and filled, on average, 4/5 of the bone defect in a centripetal direction, surrounded by the biomaterial ( Fig. 2C ). The microspheres near the edges were surrounded by the newly formed osteoid matrix, with many active osteoblasts, osteocytes, and some concentric lamellae ( Fig. 2D ). In the residual area, the formation of connective tissue with a denser appearance than the previous biological point was noted, with the presence of blood vessels and a mild chronic granulomatous inflammatory response.

**Fig. 3:**
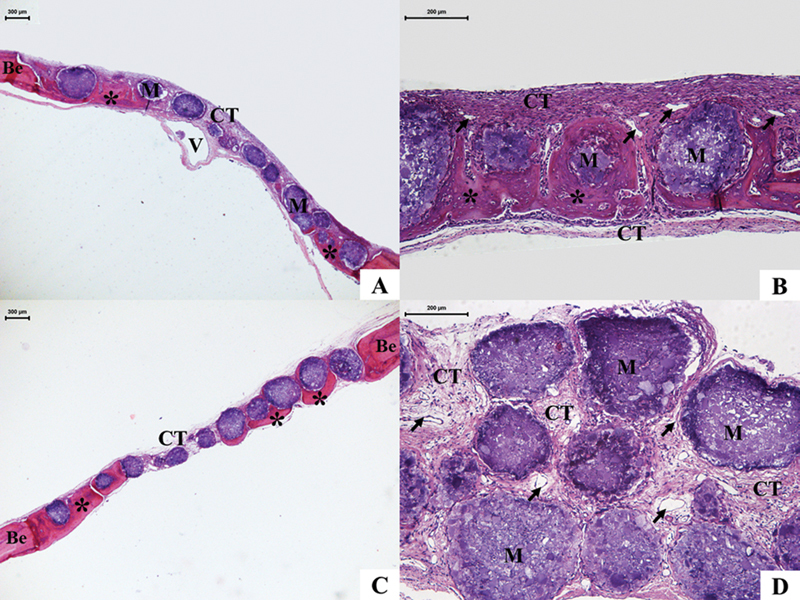
Photomicrograph of HAMgG – 15 days and 45 days. Microspheres (M) are observed distributed in a monolayer, permeated by a newly formed osteoid matrix (*), in a centripetal direction, and by connective tissue (CT), rich in blood vessels (black arrow). Abbreviations: Be, bone edge; V, central vein; HE, hematoxylin and eosin. 15 days ( **A** , **B** ). 45 days ( **C** , **D** ).

In the HAMgG, at 15 days, with the biomaterials, neoformation of the osteoid matrix was noted in a centripetal direction, more evident in the peripheral region of the defect ( Fig. 3A ). Like HA, the biomaterials filled the entire bone defect, with a thickness proportional to the edges, and arranged in a monolayer. Active osteoblasts and numerous osteocytes were observed in association with the osteoid matrix. The remaining areas were filled with loose connective tissue full of blood vessels ( Fig. 3B ) and chronic granulomatous inflammatory infiltrate of moderate appearance, mainly surrounding the microspheres.

At the biological point of 45 days, in the HAMgG, the new bone formation permeated by the microspheres extended centripetally and confluently, filling approximately 2/3 of the linear extension of the defect ( Fig. 3C ). Furthermore, it was noted that the microspheres located close to the edges were surrounded by osteoid neoformation rich in osteocytes. In the residual area, there was a new formation of connective tissue, more organized than at 15 days, and a large number of blood capillaries ( Fig. 3D ).

**Fig. 4:**
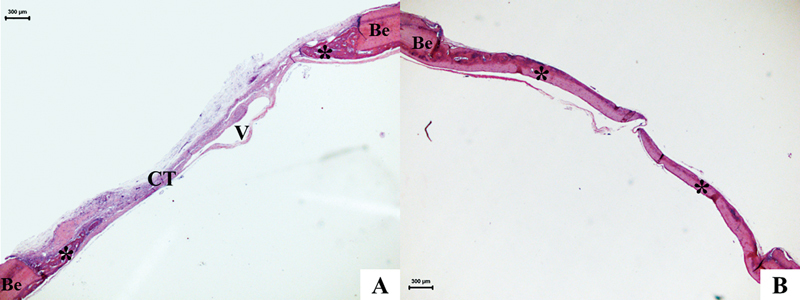
Photomicrograph of CG – 15 and 45 days. Note the neoformation of the osteoid matrix (*) in a centripetal direction, with reduced thickness about the bone edges (Be), with a residual area filled with connective tissue (CT). HE, hematoxylin and eosin. 15 days ( **A** ) and 45 days ( **B** ).

The CG, at 15 days, displayed bone neoformation with a reactional appearance associated with the edges, presenting active osteoblasts and osteocytes. The remaining area showed deposition of loose connective tissue, with reduced thickness, abundant in spindle cells and blood vessels ( Fig. 4A ). At 45 days, new bone formation extended beyond the edge in a centripetal direction, more evidently than at 15 days ( Fig. 4B ). The chronic inflammation observed was discreet and scarce. Tissue repair was completed with the formation of connective tissue in the region and no mineralization.

### Histomorphometric Analysis

The histomorphometric study displayed no statistically significant differences among the three groups evaluated at 15 days when analyzing the newly formed osteoid matrix area. At the biological point of 45 days, statistical significance was noted when comparing the HAMgG and HAG with the CG, with the latter group having a higher average of newly formed osteoid matrix ( Table 1 ).

**Table 1:** Percentage of the newly formed mineralized area in relation to the total area of the defect at the 15- and 45-days biological points, in the 3 experimental groups

Group Period	HAMgG	HAG	CG	*P-value* (between-group)	Comparison HAMgG x HAG	Comparison of CG with HAMgG and HAG
**15 days**	25% (±29)	12% (±7)	20% (±38)	*p* = 0.58 NS	*p* = 0.55 NS	HAMgG: *p* = 0.89 NS HAG: *p* = 0.82 NS
**45 days**	20% (±13)	25% (±11)	61% (±21)	*p* = 0.01 S	*p =* 0.92 NS	HAMgG: *p* = 0.01 S HAG: *p* = 0.02 S

**Abbreviations:** CG, control group; HAG, group with defect filled with hydroxyapatite microspheres; HAMgG, group with bone defect filled with magnesium-substituted hydroxyapatite microspheres group; NS, not statistically significant; S, statistically significant.

## Discussion

Experimental models in vivo have been referenced for many decades in different types of studies in various knowledge areas. To evaluate biomaterials designed for bone regeneration, these models must present anatomical and physiological characteristics that enable an understanding of natural phenomena to determine the osteogenic potential of the materials investigated. Thus, among the different possibilities for studies, the bone defect created in the calvaria of a rat stands out, which presents easy access and surgical manipulation, low cost, good predictability, and reproducibility. 14 Therefore, this study evaluated the repair of non-critical bone defects after implantation of HA microspheres substituted with Mg.

The surgical procedure to create this defect promoted tissue damage and, consequently, vascular rupture that resulted in blood leakage and clot formation, followed by release of cytokines that triggered an inflammatory response. This inflammation, chronic granulomatous, discrete, and regressive throughout the study, agreed with what occurs when a biomaterial is implanted *in vivo* . 15 16 These findings are in line with what was observed by Santos et al *.* , 2 Santos et al., 4 Miguel et al *.* , 12 Daltro et al., 13 Almeida et al. 17 and Ribeiro et al. 18 These authors evaluated different types of biomaterials for bone regeneration, in rat calvaria, and described the same tissue response.

During the physiological events that occur in bone regeneration, besides the release of cytokines and chemical mediators, there is the secretion of growth, cellular differentiation, and angiogenic factors, which stimulate the formation of connective tissue rich in new blood vessels, with consequent development of granulation tissue, as well as deposition of the osteoid matrix, which subsequently becomes mineralized. At all biological points, such histomorphological characteristics were observed in the three groups evaluated in our study. At 45 days, in the CG, the neoformation of mineralized tissue was approximately 60%. However, in HAG and HAMgG this percentage was 25 and 20%, respectively. This difference in bone neoformation in the two groups with biomaterial implantation, as compared to the CG, derives from the presence of microspheres in the bone defect since these were sintered and, consequently, not reabsorbed due to the sintering of the material – a procedure that promotes changes in the HA crystal lattice with crystal fusion. 19 Thus, in HAG and HAMgG, the microspheres occupied a large part of the sectional area of the defect and, thus, formed a three-dimensional framework throughout the linear extension and height of the bone defect.

It is worth highlighting that although some authors suggest and defend some methodologies as standard, there is still no consensus in the literature on which bone defect exact diameter shall be considered non-critical or critical. A “critical” bone defect has been defined as a bone defect that does not regenerate throughout the life of the animal 20 or of the study. 21 In these cases, the repair is completed by fibrosis, and new bone formation is restricted to the edges. 4 12 17 18 This situation was not observed in the present study, in which new mineralized tissue formed centripetally along the biological points, with regeneration of almost the entire defect area in the CG at 45 days. These findings contrast with the results obtained by authors who classify this 5.0-mm bone defect as critical. 22 23 24

Concerning bone regeneration, a 3D scaffold is essential for the cellular and vascular events observed during this mechanism. Therefore, ceramic biomaterials have been widely studied for this purpose in different forms of presentation and composition. Scientific and technological advances experienced in recent decades have increasingly enabled the development of materials with biomimetic physicochemical characteristics to stimulate new bone formation. Among these materials, HA has been extensively researched due to its biocompatibility, similarity with natural components of the mineral phase of bone tissue, osteoconductivity, bioactivity, 1 3 and lack of toxicity and immunogenicity. Our study observed such characteristics in the two groups in which biomaterials were implanted, in the three biological points, which allowed the formation of an osteoid matrix and connective tissue between and surrounding the microspheres.

The HA hexagonal structure performs ionic exchanges at the Ca ^2+^ and HPO _4_^3−^ sites with elements or functional groups that are naturally part of the bone composition to promote changes in crystallinity, solubility, biodegradation, and, consequently, in biological properties of the biomaterial after in vivo implantation. 4 25 26 Mg stands out, playing a critical role in bone metabolism and growth: the deficiency of this element inhibits the osteoblasts' activity, favoring the osteoclasts' survival and performance. 27 28 Furthermore, the presence of Mg contributes to biomineralization, mainly in the initial phase of osteogenesis: it increases the kinetics of HA nucleation, delays its crystallization, and can interfere with qualitative changes in the bone matrix. 29 This is probably why the average bone formation at 15 days was higher in HAMgG compared to HAG, however, without statistically significant differences. Despite this, this pattern did not repeat after 45 days, and the average osteoid matrix formation was similar between these groups.

Mg also has an essential effect on promoting angiogenesis, stimulating endothelial cells, and the production of vascular growth factors, 30 which may justify the presence of more evident blood vessels in HAMgG than in the other groups throughout the study. Furthermore, incorporating this metal into the HA structure promoted a slight decrease in the biomaterial crystallinity without hampering the ceramic biodegradation, probably due to sintering.

The biocompatibility and bioactivity of the biomaterials studied here demonstrate that these materials have future clinical applications, especially as filler biomaterials. Given the results obtained in this work, new studies shall analyze these biomaterials without heat treatment (sintering) and with other variations in Mg concentration in critical (8.0 mm) and non-critical defects (5.0 mm) to better characterize the osteogenic potential of these ceramics replaced with this metal.

## Conclusions

The biomaterials evaluated in this study are biocompatible, osteoconductive, and bioactive. The Mg substituted in HA stimulated a higher bone formation only in the initial phase of bone repair (15 days), forming the osteoid matrix similarly between ceramics in the final stage of the study.
