# Supplementary material for: Histomorphometric Study of Non-critical Bone Defect Repair after Implantation of Magnesium-substituted Hydroxyapatite Microspheres
Source: Rev Bras Ortop (Sao Paulo). 2024 Sep 4;59(4):e519–25. doi: 10.1055/s-0044-1787768 (PMC11374412; doi:10.1055/s-0044-1787768)
Supplement: Related texts

# Research article: Estudo histomorfométrico do reparo de defeito ósseo não crítico após implantação de microesferas de hidroxiapatita com substituição por magnésio

**Keywords:** biomateriais, hidroxiapatitas, magnésio, osso e ossos, regeneração óssea

## Abstract

**Objetivo** O presente estudo teve como objetivo fazer uma análise histomorfométrica do reparo de um defeito ósseo não crítico após a implantação de microesferas de hidroxiapatita (HA) com substituição por magnésio (Mg).

**Métodos** Trinta ratos foram distribuídos em 3 grupos experimentais, avaliados aos 15 e 45 dias após a cirurgia: GHA (defeito ósseo preenchido com microesferas de HA); GHAMg (defeito ósseo preenchido com microesferas de HA com substituição por 1 mol% de Mg) e GC (defeito ósseo sem implantação de biomateriais).

**Resultados** Aos 15 dias, evidenciou-se que os biomateriais preencheram toda a extensão do defeito, com neoformação de matriz osteoide de permeio às microesferas. No GC, essa neoformação ficou restrita às bordas, com deposição de tecido conjuntivo frouxo de espessura reduzida. Aos 45 dias, a neoformação óssea preencheu quase toda a extensão do defeito ósseo nos 3 grupos, com deposição osteoide estatisticamente significativa no GC, apesar da espessura reduzida em comparação ao GHA e o GHAMg. Os grupos com implantação de biomaterial apresentaram matriz osteoide mais abundante do que aos 15 dias.

**Conclusão** Os biomateriais estudados apresentaram biocompatibilidade, osteocondutividade e bioatividade. A concentração de Mg na HA com substituição não estimulou a formação óssea mais significativa do que a HA sem este íon.

## Introdução

A bioengenharia tecidual óssea, área emergente, interdisciplinar e multidisciplinar, tem ganhado destaque nos últimos anos devido aos avanços técnico-científicos alcançados e as crescentes demandas biomédicas e socioeconômicas da sociedade atual. Os investigadores desta área têm aplicado os princípios das ciências biológicas e da saúde, da química, da física e da engenharia para desenvolver e melhorar técnicas regenerativas e biomateriais capazes de restaurar ou melhorar a função e a estética de tecidos e órgãos comprometidos. 1 2 3 4

A aplicabilidade desses biomateriais é definida pela presença de propriedades físico-químicas, biológicas e morfológicas semelhantes e compatíveis com tecidos vivos para atuarem como arcabouços tridimensionais (3D), que auxiliem a regeneração tecidual ou, ainda, atuem como substitutos adequados de tecidos e órgãos danificados ou perdidos. Dentre os diversos biomateriais hoje disponíveis, as biocerâmicas de fosfato de cálcio (CaP) representam uma classe amplamente pesquisada e utilizada em técnicas de regeneração óssea. 1 Dentre esses materiais, a hidroxiapatita (HA) sintética tem se destacado nas últimas décadas devido à sua biocompatibilidade, semelhança com a apatita biológica, bioatividade, osteocondutividade, não imunogenicidade e eventos celulares facilitadores observados durante a regeneração tecidual, como angiogênese e osteogênese. 1 2 3 4 No entanto, esta cerâmica apresenta baixas taxas de biodegradação e bioabsorção após a implantação *in vivo* , assíncronas em relação ao mecanismo de regeneração óssea. 1 2 3 4 Além disso, é um material rígido e quebradiço, que pode permanecer no sítio de implantação por meses e até anos, dependendo do método de síntese e processamento. 4 5

Diante disso, um dos principais objetivos dos pesquisadores nesta área tem sido melhorar as propriedades da HA sintética e modificar as características deste material para potencializar a resposta tecidual após a implantação *in vivo* . Dentre os diferentes métodos disponíveis para realizar essas modificações, as substituições isomórficas da estrutura hexagonal da HA têm apresentado resultados satisfatórios 3 6 devido aos efeitos nas propriedades físicas do material, observados por alterações nos parâmetros de rede, estrutura cristalina, morfologia, solubilidade e estabilidade térmica em comparação à HA sem substituição. 7

Nestes casos, outros metais, como o zinco (Zn), estrôncio (Sr), flúor (F), manganês (Mn) e magnésio (Mg), podem substituir o cálcio (Ca). 3 4 6 O magnésio (Mg) tem despertado grande interesse científico, visto que, entre outras propriedades, participa da homeostase do tecido ósseo. Além disso, juntamente com outros minerais, é fundamental para o mecanismo de regeneração óssea, pois estimula a formação óssea por meio da ativação de osteoblastos, e inibe a reabsorção por sua ação sobre os osteoclastos. 8 9 10 11

Apesar dessas possibilidades, as propriedades físico-químicas, o comportamento biológico e a capacidade regenerativa do CaP substituído por Mg ainda necessitam de mais estudos para assegurar a eficácia das técnicas utilizadas durante a síntese e processamento desses biomateriais. Portanto, este estudo avaliou o reparo de defeitos ósseos não críticos após a implantação de microesferas de HA com substituição por Mg na calvária de ratos.

## Materiais e Métodos

## Biomateriais

Os biomateriais avaliados neste estudo foram sintetizados, processados e esterilizados no Laboratório de Biomateriais (LABIOMAT) do Centro Brasileiro de Pesquisas Físicas (CBPF). Sua síntese, processamento e caracterização foram descritos por Santos et al. 4

## Procedimentos Cirúrgicos

Este estudo foi realizado no biotério da Universidade Estadual de Feira de Santana (UEFS) após aprovação do Comitê de Ética no Uso de Animais (CEUA) da Escola Bahiana de Medicina e Saúde Pública, protocolo 02/2013. Trinta ratos Wistar adultos, machos, com peso corporal entre 350 e 400 g, foram distribuídos aleatoriamente em 3 grupos experimentais com 5 animais cada: GHA (grupo com defeito preenchido com microesferas de HA); GHAMg (grupo com defeito ósseo preenchido com microesferas de MgHA) e GC (grupo controle com defeito ósseo sem implante de biomaterial). Os animais foram avaliados 15 e 45 dias após a cirurgia. A técnica cirúrgica utilizada foi a mesma descrita por Miguel et al. 12 e ilustrada por Santos et al. 2 Porém, vale ressaltar que, nestes estudos, o defeito ósseo tinha aproximadamente 8,0 mm de diâmetro, mas aqui tinha cerca de 5,0 mm 13 ( Fig. 1 ).

**Fig. 1:** Etapas cirúrgicas para criação de um defeito ósseo não crítico. ( **A** ) Região da calvária após tricotomia e antissepsia; ( **B** ) retalho rebatido após incisão semilunar bicoronal na pele para exposição do tecido ósseo; ( **C** ) demarcação do defeito ósseo na porção mediana da calvária; e ( **D** ) defeito ósseo não crítico criado.

## Processamento Histológico e Análise Histomorfológica

Nos pontos biológicos de 15 e 45 dias, os animais foram eutanasiados com dose letal de cetamina e xilazina. Em seguida, a porção superior da calvária foi removida, os tecidos moles foram descartados e os espécimes foram fixados em formaldeído tamponado a 4% por 7 dias. Após esta etapa, foram descalcificados em ácido nítrico 5% por 2 horas, embebidos em parafina e cortados com 5,0 μm de espessura. Os cortes histológicos obtidos foram corados com hematoxilina-eosina (HE) e posteriormente examinados em microscopia óptica padrão (DM1000–Leica Microsystems, Wetzlar, Alemanha) para análise histomorfológica e morfométrica. Uma câmera digital (DFC 310 FX – Leica Camera AG, Wetzlar, Alemanha) acoplada a um microscópio óptico comum (DM1000–Leica) e ao software QWin 3.1 (Leica) mediu a área da matriz mineralizada recém-formada nos 3 grupos experimentais. Posteriormente, foi realizada análise estatística com base na média e no desvio-padrão para obtenção do valor de *p* , com nível de significância de 5% ( *p* < 0,05), por meio de análise de variância (ANOVA).

## Resultados

## Análise Histomorfológica

Aos 15 dias, o GHA apresentou neoformação de matriz osteoide associada às bordas ósseas e de modo circunjacente à algumas microesferas, que se organizavam predominantemente em monocamada e ocupavam toda a extensão do defeito ósseo, mantendo a espessura proporcional às bordas ( Fig. 2A ). Osteócitos e osteoblastos ativos estavam associados a esta matriz. Observou-se formação de tecido conjuntivo e inflamação crônica discreta com infiltrado inflamatório mononuclear e células gigantes multinucleadas, principalmente ao redor das microesferas, entre as áreas mineralizadas e as microesferas ( Fig. 2B ).

**Fig. 2:** Fotomicrografia do grupo com defeito preenchido com microesferas de hidroxiapatita (GHA) – 15 e 45 dias. Notar as microesferas (M) distribuídas em camada única ou dupla, com formação de matriz osteoide (*) em quase toda a extensão do defeito, com presença de osteócitos (OCs) e organizadas em lamelas ósseas concêntricas; tecido conjuntivo (CT) frouxo na área residual do defeito ósseo, com presença de células inflamatórias (Ci) mononucleares e células gigantes multinucleadas (seta amarela). Abreviaturas: Be, borda óssea; HE, hematoxilina-eosina. 15 dias ( **A** , **B** ). 45 dias ( **C** , **D** ).

No ponto biológico de 45 dias, as microesferas no GHA estavam distribuídas principalmente em multicamadas. A matriz osteoide neoformada ficou mais evidente que aos 15 dias e preencheu, em média, 4/5 do defeito ósseo no sentido centrípeto, circundado pelo biomaterial ( Fig. 2C ). As microesferas próximas às bordas estavam circundadas pela matriz osteoide recém-formada, com muitos osteoblastos ativos, osteócitos e algumas lamelas concêntricas ( Fig. 2D ). Na área residual, notou-se a formação de tecido conjuntivo de aspecto mais denso que no ponto biológico anterior, com presença de vasos sanguíneos e resposta inflamatória granulomatosa crônica discreta.

No GHAMg, aos 15 dias, com os biomateriais, notou-se neoformação da matriz osteoide no sentido centrípeto, mais evidente na região periférica do defeito ( Fig. 3A ). Assim como no GHA, os biomateriais preencheram todo o defeito ósseo, com espessura proporcional às bordas, e dispostos em monocamada. Osteoblastos ativos e numerosos osteócitos foram observados em associação à matriz osteoide. As demais áreas estavam preenchidas por tecido conjuntivo frouxo repleto de vasos sanguíneos ( Fig. 3B ) e infiltrado inflamatório granulomatoso crônico de aspecto moderado, principalmente circundando as microesferas.

**Fig. 3:** Fotomicrografia do grupo com defeito ósseo preenchido com microesferas de hidroxiapatita substituída por magnésio (GHAMg) – 15 dias e 45 dias. Observam-se microesferas (M) distribuídas em monocamada, permeadas por matriz osteoide recém-formada (*), em sentido centrípeto, e por tecido conjuntivo (CT), rico em vasos sanguíneos (seta preta). Abreviaturas: BE, borda óssea; V, veia central; HE, hematoxilina-eosina. 15 dias ( **A** , **B** ). 45 dias ( **C** , **D** ).

No ponto biológico de 45 dias no GHAMg, a neoformação óssea permeada pelas microesferas estendeu-se de forma centrípeta e confluente, preenchendo aproximadamente 2/3 da extensão linear do defeito ( Fig. 3C ). Além disso, notou-se que as microesferas localizadas próximas às bordas estavam circundadas por neoformação osteoide rica em osteócitos. Na área residual, houve nova formação de tecido conjuntivo, mais organizado que aos 15 dias, e grande número de capilares sanguíneos ( Fig. 3D ).

O GC, aos 15 dias, apresentou neoformação óssea com aspecto reacional associado às bordas, com osteoblastos e osteócitos ativos. Na área remanescente, notou-se deposição de tecido conjuntivo frouxo, com espessura reduzida, abundante em células fusiformes e vasos sanguíneos ( Fig. 4A ). Aos 45 dias, a nova formação óssea estendeu-se além da borda em direção centrípeta, mais evidentemente do que aos 15 dias ( Fig. 4B ). A inflamação crônica observada foi discreta e escassa. A reparação tecidual foi completada com formação de tecido conjuntivo na região e ausência de mineralização.

**Fig. 4:** Fotomicrografia do grupo controle (GC) – 15 e 45 dias. Note a neoformação da matriz osteoide (*) em direção centrípeta, com espessura reduzida nas bordas ósseas (BEs) e área residual preenchida por tecido conjuntivo (CT). Abreviatura: HE, hematoxilina-eosina. 15 dias ( **A** ) e 45 dias ( **B** ).

## Análise Histomorfométrica

O estudo histomorfométrico não apresentou diferenças estatisticamente significativas entre os 3 grupos avaliados aos 15 dias ao analisar a área da matriz osteoide recém-formada. No ponto biológico de 45 dias, notou-se significância estatística ao comparar o GHAMg e o GHA com o GC, sendo que este último grupo apresentou maior média de matriz osteoide recém-formada ( Tabela 1 ).

**Tabela 1:** Porcentagem da área mineralizada neoformada em relação à área total do defeito nos pontos biológicos de 15 e 45 dias nos 3 grupos experimentais

Grupo Tempo	GHAMg	GHA	GC	Valor de *p* (entre grupos)	Comparação GHAMg x GHA	Comparação de GC com GHAMg e GHA
**15 dias**	25% (±29)	12% (±7)	20% (±38)	*p* = 0,58 NS	*p* = 0,55 NS	GHAMg: *p* = 0,89 NS GHA: *p* = 0,82 NS
**45 dias**	20% (±13)	25% (±11)	61% (±21)	*p* = 0,01 S	*p =* 0,92 NS	GHAMg: *p* = 0,01 S GHA: *p* = 0,02 S

**Abreviaturas:** GC, grupo controle; GHA, grupo com defeito preenchido com microesferas de hidroxiapatita; GHAMg, grupo com defeito ósseo preenchido com microesferas de hidroxiapatita substituída por magnésio; NS, não estatisticamente significante; S, estatisticamente significante.

## Discussão

Modelos experimentais *in vivo* são referência há muitas décadas em diferentes tipos de estudos e diversas áreas do conhecimento. Para avaliar biomateriais destinados à regeneração óssea, esses modelos devem apresentar características anatômicas e fisiológicas que possibilitem a compreensão dos fenômenos naturais para determinar o potencial osteogênico dos materiais investigados. Assim, dentre as diferentes possibilidades de estudos, destaca-se o defeito ósseo criado na calvária de ratos, que apresenta acesso e manipulação cirúrgica fáceis, baixo custo e boa previsibilidade e reprodutibilidade. 14 Portanto, este estudo avaliou o reparo de defeitos ósseos não críticos após implantação de microesferas de HA com substituição por Mg.

O procedimento cirúrgico para criação desse defeito promoveu dano tecidual e, consequentemente, ruptura vascular que provocou extravasamento sanguíneo e formação de coágulos, seguida de liberação de citocinas que desencadearam resposta inflamatória. Essa inflamação granulomatosa crônica, discreta e regressiva ao longo do estudo, está de acordo com o que ocorre após a implantação *in vivo* de um biomaterial. 15 16 Esses achados estão de acordo com aqueles observados por Santos et al., 2 Santos et al., 4 Miguel et al., 12 Daltro et al., 13 Almeida et al. 17 e Ribeiro et al. 18 Esses autores avaliaram diferentes tipos de biomateriais para regeneração óssea em calvária de ratos e descreveram a mesma resposta tecidual.

Durante os eventos fisiológicos que ocorrem na regeneração óssea, além da liberação de citocinas e mediadores químicos, há a secreção de fatores de crescimento, diferenciação celular e angiogênicos que estimulam a formação de tecido conjuntivo rico em novos vasos sanguíneos, com consequente desenvolvimento de tecido de granulação e deposição de matriz osteoide, que posteriormente se torna mineralizada. Em todos os pontos biológicos, tais características histomorfológicas foram observadas nos três grupos avaliados em nosso estudo. Aos 45 dias, no GC, a neoformação de tecido mineralizado foi de aproximadamente 60%. Porém, no GHA e no GHAMg, esse percentual foi de 25 e 20%, respectivamente. Essa diferença na neoformação óssea nos dois grupos com implantação de biomaterial em relação ao GC decorre da presença de microesferas no defeito ósseo, uma vez que estas foram sinterizadas e, consequentemente, não reabsorvidas devido à sinterização do material, um procedimento que promove alterações na rede cristalina da HA com fusão dos cristais. 19 Assim, nos grupos GHA e GHAMg, as microesferas ocuparam grande parte da área seccional do defeito e formaram uma estrutura tridimensional ao longo da extensão linear e altura do defeito ósseo.

Vale ressaltar que embora alguns autores sugiram e defendam algumas metodologias como padrão, ainda não há consenso na literatura sobre qual diâmetro exato do defeito ósseo deve ser considerado não crítico ou crítico. Um defeito ósseo “crítico” foi definido como aquele que não se regenera ao longo da vida do animal 20 ou do estudo. 21 Nestes casos, o reparo é completado por fibrose e a nova formação óssea fica restrita às bordas. 4 12 17 18 Essa situação não foi observada no presente estudo, em que o novo tecido mineralizado se formou de maneira centrípeta ao longo dos pontos biológicos, com regeneração de quase toda a área do defeito no GC aos 45 dias. Esses achados contrastam com os obtidos por autores que classificam esse defeito ósseo de 5,0 mm como crítico. 22 23 24

No que se refere à regeneração óssea, um arcabouço tridimensional (3D) é essencial para os eventos celulares e vasculares observados durante este mecanismo. Portanto, biomateriais cerâmicos têm sido amplamente estudados para esse fim em diferentes formas de apresentação e composição. Os avanços científicos e tecnológicos vivenciados nas últimas décadas têm possibilitado cada vez mais o desenvolvimento de materiais com características físico-químicas biomiméticas voltadas para a estimular a neoformação óssea. Dentre esses materiais, a HA tem sido amplamente pesquisada devido à sua biocompatibilidade, semelhança com componentes naturais da fase mineral do tecido ósseo, osteocondutividade, bioatividade 1 3 e ausência de toxicidade e imunogenicidade. Nosso estudo observou tais características nos dois grupos submetidos à implantação dos biomateriais, nos dois pontos biológicos, o que permitiu a formação de uma matriz osteoide e tecido conjuntivo entre e ao redor das microesferas.

A estrutura hexagonal da HA realiza trocas iônicas nos sítios Ca ^2+^ e HPO _4_^3−^ com elementos ou grupos funcionais que fazem parte naturalmente da composição óssea para alterar a cristalinidade, solubilidade, biodegradação e, consequentemente, as propriedades biológicas do biomaterial após a implantação *in vivo* . 4 25 26 O Mg se destaca por desempenhar um papel fundamental no metabolismo e crescimento ósseo: a deficiência desse elemento inibe a atividade dos osteoblastos, enquanto favorece a sobrevida e a atuação dos osteoclastos. 27 28 Além disso, a presença de Mg contribui para a biomineralização, principalmente na fase inicial da osteogênese: aumenta a cinética de nucleação da HA, retarda sua cristalização e pode interferir nas alterações qualitativas da matriz óssea. 29 Isso provavelmente explica por que a média de formação óssea aos 15 dias foi maior no GHAMg do que no GHA, mas sem diferenças estatisticamente significativas. Apesar disso, esse padrão não se repetiu após 45 dias e a formação média de matriz osteoide foi semelhante entre esses grupos.

O Mg também tem efeito essencial na promoção da angiogênese, na estimulação das células endoteliais e na produção de fatores de crescimento vascular, 30 o que pode justificar a presença de vasos sanguíneos mais evidentes no GHAMg do que nos demais grupos ao longo do estudo. Além disso, a incorporação deste metal na estrutura da HA promoveu uma ligeira diminuição na cristalinidade do biomaterial sem prejudicar a biodegradação da cerâmica, provavelmente devido à sinterização.

A biocompatibilidade e bioatividade dos biomateriais aqui estudados demonstram que esses materiais podem ser utilizados em futuras aplicações clínicas, em especial como biomaterial de preenchimento. Diante dos resultados obtidos neste trabalho, novos estudos deverão analisar estes biomateriais sem tratamento térmico (sinterização) e com outras variações na concentração de Mg em defeitos críticos (8,0 mm) e não críticos (5,0 mm) para melhor caracterizar o potencial osteogênico destas cerâmicas com substituição por este metal.

## Conclusões

Os biomateriais avaliados neste estudo são biocompatíveis, osteocondutores e bioativos. O Mg substituído na HA estimulou a maior formação óssea apenas na fase inicial do reparo ósseo (15 dias), com formação de matriz osteoide de forma semelhante entre as cerâmicas na fase final do estudo.
